# Isolation and Functional Characterization of the MADS-Box Gene *AGAMOUS-LIKE 24* in Rubber Dandelion (*Taraxacum kok-saghyz* Rodin)

**DOI:** 10.3390/ijms26052271

**Published:** 2025-03-04

**Authors:** Yijiao Cai, Wei Yang, Jin Yue, Jiaqi Chen, Jianfeng Xing, Xue Yang, De Ye, Chaorong Tang, Hui Liu

**Affiliations:** 1 School of Breeding and Multiplication (Sanya Institute of Breeding and Multiplication), Hainan University, Sanya 572025, China; 2 School of Tropical Agriculture and Forestry, Hainan University, Sanya 572025, China; 3 National Key Laboratory for Biological Breeding of Tropical Crops, Hainan University, Haikou 570228, China; 4 Natural Rubber Cooperative Innovation Center of Hainan Province and Ministry of Education of PRC, Hainan University, Haikou 570228, China

**Keywords:** AGL24, rubber dandelion, floral induction, flower development, MADS-box gene, natural rubber production

## Abstract

Rubber dandelion (*Taraxacum kok-saghyz* Rodin, TKS), also referred to as Russian dandelion, is one of the most promising natural rubber (NR)-producing plants that produce high-quality NR comparable to that from the Pará rubber tree (*Hevea brasiliensis*, Hb), currently the only commercial source. It needs further breeding to improve the agricultural traits. However, little has been known about the genetic mechanisms underlying the regulation of floral induction and flower development in TKS, an important trait that remains to be improved for commercial production. The MADS-box gene *AGAMOUS-LIKE 24* (*AGL24*) plays important roles in floral induction and flower development. As the first step in understanding its roles in TKS, this study isolated and characterized the *AGL24*-homologous gene *TkAGL24* in TKS. The *TkAGL24* gene had a 705 bp coding sequence (CDS) that encoded a protein of 234 amino acids containing the conserved classic MADS-box type II domain and K-box domain, sharing 55.32% protein sequence identity with the AtAGL24 protein from *Arabidopsis*. *TkAGL24* was highly expressed in leaf, latex, root, and peduncle but rarely or not in mature flower. The TkAGL24 protein was located in the nucleus and cytoplasm and did not have transcription activation activity in yeast cells. The overexpression of *TkAGL24* in *Arabidopsis* could promote flowering and cause the abnormal development of flowers, similar to other *AGL24*-homologous genes from other species. Furthermore, the overexpression of *TkAGL24* in TKS also affected the development of ligulate flowers. These results suggested that the cloned *TkAGL24* gene is functional and may play important roles in floral induction and flower development in TKS, providing an insight into the possibility for the further studies of its roles and application to breeding.

## 1. Introduction

Natural rubber (NR) is an important resource in both civil and defense industries, which is valued for its unique physicochemical properties, such as high elasticity, resilience, and tear resistance. Although NR is indispensably used in a wide range of industrial products, its commercial supply is almost exclusively from the Pará rubber tree (*Hevea brasiliensis*, Hb), which is facing significant difficulties due to the easy susceptibility to the fatal disease, being limited to tropical plantation conditions, and high labor costs. Therefore, considerable research attention has been drawn to the development of new alternative NR-producing plants [1]. Among them, the rubber dandelion (*Taraxacum kok-saghyz* Rodin, TKS), also referred to as the Russian dandelion, is emerging as one of the most promising alternative NR-producing plants [2,3], which could produce more than 20% high-quality NR on the basis of dry root weight [4]. Moreover, TKS can be cultivated much more widely in most temperate regions, compared to the tropic-limited range of Hb plantation [5]. In addition, the roots of TKS also produce inulin, an industrially valuable carbohydrate, up to 40% of the root dry weight [6,7]. Taken together, TKS has great potential as an alternative source of NR and other economically important metabolites. However, further breeding and domestication are required before TKS can be used as an industrial crop.

TKS is a perennial herbaceous plant belonging to the genus *Taraxacum* within the Asteraceae family [8]. Previous studies have demonstrated that the significant yields of NR and inulin were highly related to plant maturation, which could be associated with floral induction [4,9,10]. On the other hand, floral induction and flower development are also important for breeding and domestication. However, the regulatory mechanisms underlying floral induction and flower development in TKS remain poorly understood.

In the model plant *Arabidopsis*, the *AGAMOUS-LIKE 24* (*AtAGL24*) gene from the MADS-box gene family has been demonstrated to be a key transcription factor in floral transition [11,12,13]. *AGL24* and *SHORT VEGETATIVE PHASE* (*SVP*) are two paralogs within the *SVP*-like subfamily, but they exhibit diametrically opposite functions during floral transition. SVP acts as a flowering repressor, while AGL24 accelerates flowering by interacting with the SUPPRESSOR OF OVEREXPRESSION OF CONSTANS1 (SOC1) to activate the expression of *LEAFY* (*LFY*) [14,15,16]. *AtAGL24* is highly expressed in the inflorescence meristem. Its expression is further upregulated by vernalization [13]. Moreover, AGL24 also positively regulates inflorescence identity and the development of floral organs [17,18]. The overexpression of *AGL24* in *Arabidopsis* could cause abnormal floral organs, including green petals, elongated carpels, and decreased fertility [13]. Therefore, *AGL24* plays important roles in floral development.

To elucidate the genetic mechanisms underlying floral induction and flower development in TKS, we identified and characterized the *AtAGL24*-homologous gene in TKS, designated as *TkAGL24*. Our results showed that the TkAGL24 protein shared high homology with AtAGL24. It is ubiquitously expressed in various tissues, except in mature flowers, and is located in both the nucleus and cytoplasm. The overexpression of *TkAGL24* in *Arabidopsis* promoted flowering and caused morphological changes in floral organs. Similarly, the overexpression of *TkAGL24* in TKS led to aberrant ligulate flowers. All these results suggested that *TkAGL24* is a functional gene and could play important roles in floral induction and flower development in TKS.

## 2. Results

### 2.1. TkAGL24 Shared the Highest Homology with the Arabidopsis AGL24 (AtAGL24)

**Figure 1:**
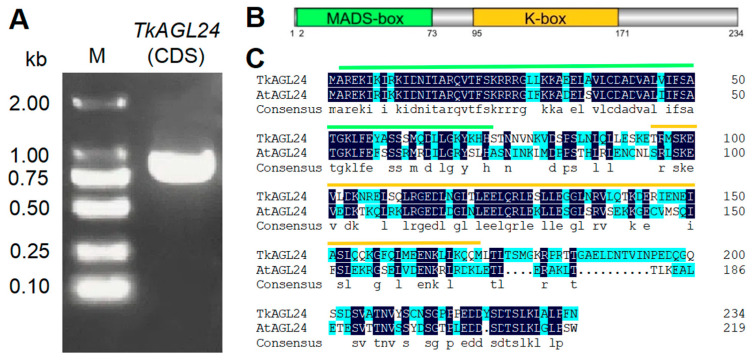
Cloning of *TkAGL24*: (**A**) Amplification of the *TkAGL24* CDS by reverse transcription polymerase chain reaction (RT-PCR). (**B**) The schematic diagram of the TkAGL24 protein. The green box indicates the MADS-box domain. The orange box indicates the K-box domain. (**C**) The amino acid sequence of the TkAGL24 protein, compared with that of the *Arabidopsis* AGL24 (AtAGL24). The green line indicates the MADS-box domain. The orange line indicates the K-box domain. Homology levels are highlighted as follows: 100% in black, and ≥50% in blue. CDS, coding sequence; kb, kilobase pairs; and M, marker.

A BLASTP search against the annotated TK20 genome database identified a protein encoded by *LG06.1290* as the closest homolog to AtAGL24. The CDS of *LG06.1290*, cloned via RT-PCR and verified by sequencing, had 705 bp in length and encoded a protein of 234 amino acids (Figure 1A,B). The protein contained a highly conserved classic MADS-box type II domain and a K-box domain (Figure 1B). Sequence alignment revealed that the protein shared the highest 55.32% of protein sequence identity with AtAGL24 (Figure 1C). Then, the *LG06.1290* gene was renamed *TkAGL24*.

### 2.2. Phylogenetic Relationship of TkAGL24 and the MADS-Box Proteins from Other Plants

**Figure 2:**
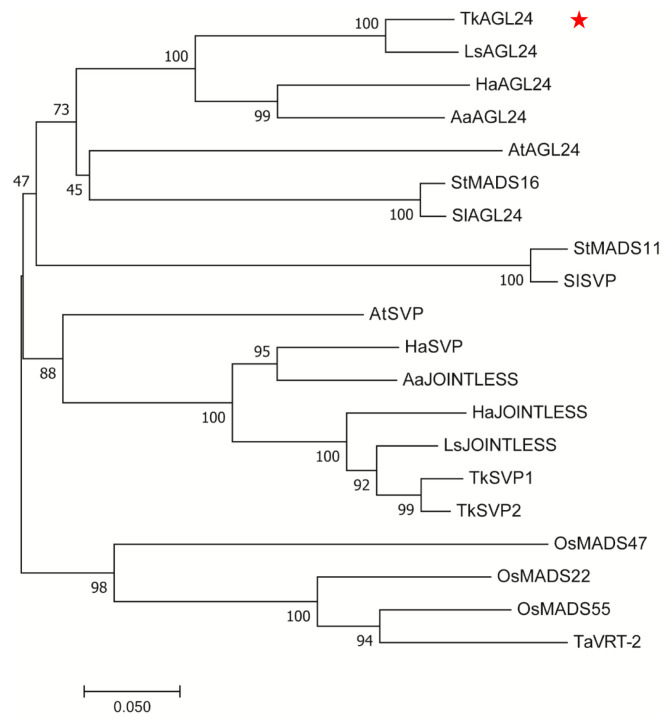
The phylogenetic relationship of TkAGL24 and the AGL24/SVP-homologous proteins from other species. The phylogenetic tree is constructed using the neighbor-joining methods under MEGA 11. Tk, *Taraxacum kok-saghyz* Rodin; At, *Arabidopsis thaliana*; Aa, *Artemisia annua*; Ha, *Helianthus annuus*; Ls, *Lactuca sativa*; Sl, *Solanum lycopersicum*; St, *Solanum tuberosum*; Os, *Oryza sativa*; and Ta, *Triticum aestivum*. The red star indicates the TkAGL24.

To further investigate the phylogenetic relationship of TkAGL24 and the MADS-box proteins from other plants, a phylogenetic tree was constructed based on the protein sequences of TkAGL24 and AGL24/SVP-homologous proteins from *Lactuca sativa* (Ls), *Helianthus annuus* (Ha), *Artemisia annua* (Aa), *Arabidopsis thaliana* (At), *Solanum lycopersicum* (Sl), *Solanum tuberosum* (St), *Oryza sativa* (Os), and *Triticum aestivum* (Ta). As shown in Figure 2, the AGL24 and SVP proteins were grouped into two separate clades. TkAGL24 clustered within the AGL24 clade and exhibited the closest relationship with LsAGL24 from lettuce (*L. sativa*), a species highly related to TKS. The *LsAGL24* has been reported to be specifically induced in a bolting-sensitive line S39, suggesting that it may have the function of regulating bolting time [19]. These results further indicated that *TkAGL24* is the ortholog of *AtAGL24* in TKS.

**Figure 3:**
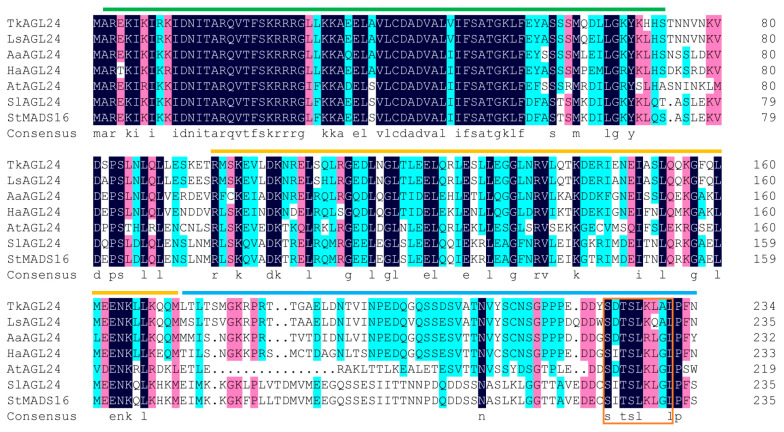
Comparison of TkAGL24 with the AGL24-homologous proteins from other species. The green line indicates the MADS-box domain. The orange line indicates the K-box domain. The blue line indicates the C-terminal domain. The orange box indicates the SDTSLKLAL motif. Homology levels are highlighted as follows: 100% in black, ≥75% in red, and ≥50% in blue. Tk, *Taraxacum kok-saghyz* Rodin; At, *Arabidopsis thaliana*; Aa, *Artemisia annua*; Ha, *Helianthus annuus*; Ls, *Lactuca sativa*; Sl, *Solanum lycopersicum*; and St, *Solanum tuberosum*.

Furthermore, the protein sequence alignment of TkAGL24 with six homologous proteins from the other species indicated that TkAGL24 shared higher sequence identities with homologous proteins from the Asteraceae family than with those from the Solanaceae and Brassicaceae families. In particular, TkAGL24 had 92.34%, 73.08%, and 71.37% protein sequence identities with LsAGL24, AaAGL24, and HaAGL24, respectively. In contrast, it shared relatively lower sequence identities of 57.74%, 56.96%, and 55.32% with the SlAGL24, StMADS16, and AtAGL24, respectively (Figure 3). In addition, the C-terminal domain sequences of AGL24 homologs varied significantly across different plant families (Figure 3).

### 2.3. TkAGL24 Was Not Induced by Vernalization

**Figure 4:**
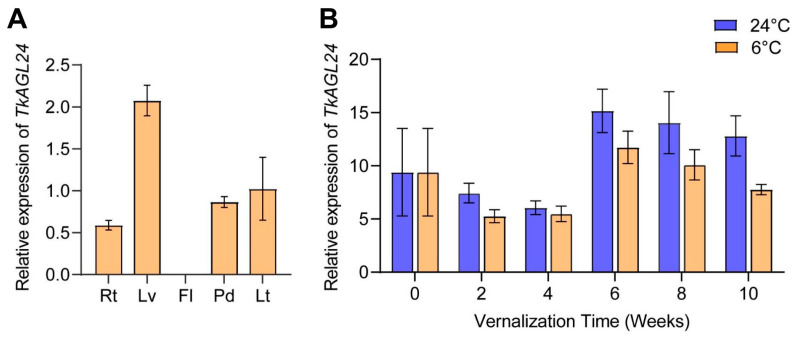
The expression profiles of *TkAGL24* in TKS: (**A**) The expression of *TkAGL24* in the roots (Rt), leaves (Lv), flowers (Fl), peduncles (Pd) and latex (Lt) of TKS, as revealed by RT-qPCR. (**B**) The impact of vernalization treatment on the expression of *TkAGL24* in the leaves of TKS. Error bars in (**A**,**B**) represent the mean ± standard deviation of triplicate experiments.

To better understand the potential roles of *TkAGL24*, its expression profile in different tissues was analyzed using RT-qPCR. The results showed that *TkAGL24* was expressed in leaves, latex, roots, and peduncles but was rarely or not expressed in the flowers (Figure 4A). In *Arabidopsis*, the expression of *AtAGL24* is significantly promoted by vernalization [13]. To examine whether vernalization could influence the expression of *TkAGL24*, its expression levels in the leaves of TK20 plants that had been vernalized for 0, 2, 4, 6, 8, and 10 weeks were detected by RT-qPCR. The results showed that the transcript abundance of *TkAGL24* only had a slight increase after six weeks of vernalization but exhibited no significant difference compared to those of the control plants grown at 24 °C (Figure 4B). Therefore, *TkAGL24* was unlikely regulated by vernalization.

### 2.4. TkAGL24 Was Located in the Nucleus and Lacked Transcription Activation Activity in Yeast Cells

**Figure 5:**
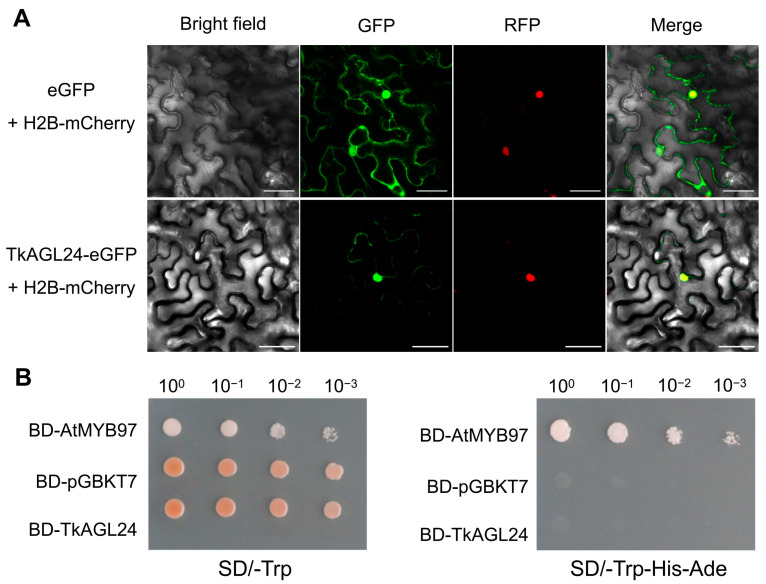
TkAGL24-eGFP is located in the nucleus and cytoplasm of the tobacco leaf cells and does not have transcription activation activity in the yeast cells: (**A**) Subcellular localization of the TkAGL24-eGFP in the tobacco leaf cells. The GFP signals are represented by green, and the mCherry signals by red. The upper panels show that control naked eGFP signals are distributed in the whole cells. The lower panels show that the TkAGL24-eGFP signals are detected much stronger in the nucleus and are significantly weaker in the cytoplasm. Bars = 50 μm. (**B**) A transcription activation assay, showing that TkAGL24 cannot activate the transcription of the reporter genes in the yeast cell.

To investigate the subcellular localization of TkAGL24, the p*35S*:*TkAGL24*-*eGFP* was co-transformed with the nuclear marker construct (p*35S*:*H2B*-*mCherry*) into the tobacco leaf cells. The results showed that TkAGL24-eGFP signals were detected in the nucleus and cytoplasm (Figure 5A). As shown in the lower panels of Figure 5A, the TkAGL24-eGFP signals in the cytoplasm were relatively weaker compared to those in the nuclei. In contrast, the naked eGFP signals from the control, which were expressed by p*35S*:*eGFP*, appeared in the whole cell, including the nucleus, likely due to the passive diffusion of eGFP into the nucleus (Figure 5A). These results demonstrated that TkAGL24 is a nucleus-localized protein.

TkAGL24 protein is a MIKC-type MADS-box transcription factor. To determine whether TkAGL24 had transcription activation activity, a yeast transcription assay was performed. The BD-TkAGL24 construct, along with the positive control construct BD-AtMYB97 and the negative control construct pGBKT7, was transformed into Y2HGold yeast cells, respectively. The resulting transformants carrying the BD-AtMYB97 construct showed vigorous growth on synthetic dropout (SD) media lacking tryptophan, histidine, and adenine. In contrast, yeast colonies transformed with either the BD-TkAGL24 construct or the empty vector pGBKT7 failed to grow (Figure 5B), indicating that TkAGL24 is unable to activate the reporter genes. These results suggest that TkAGL24 did not have transcription activation activity in the yeast test system.

### 2.5. Overexpression of TkAGL24 Promoted Flowering and Affected Floral Development in the Transgenic Arabidopsis 

**Figure 6:**
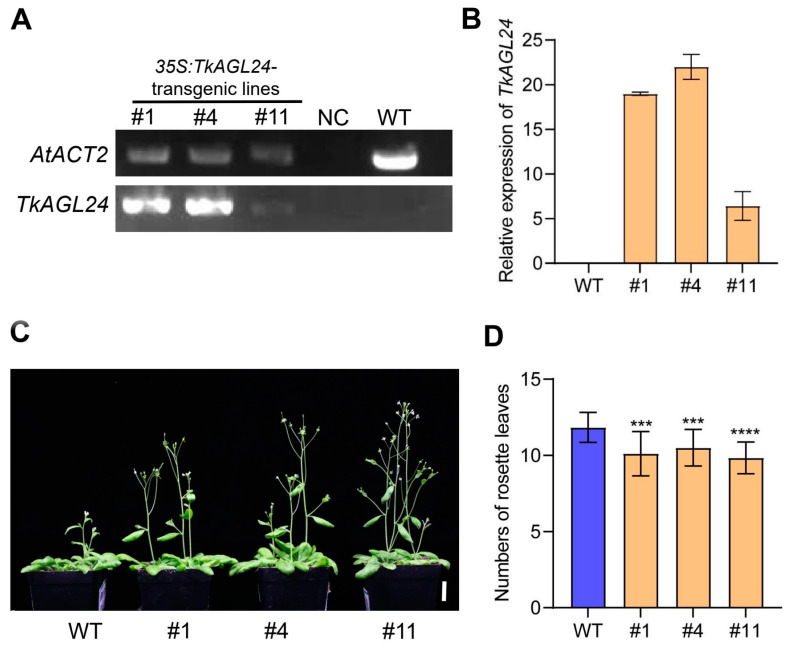
The overexpression of *TkAGL24* promotes flowering in *Arabidopsis*: (**A**,**B**) The overexpression of TkAGL24 in the transgenic lines, as revealed by RT-PCR (**A**) and RT-qPCR (**B**). Error bars in (**B**) represent the mean ± standard deviation of triplicate experiments. (**C**) The transgenic plants overexpressing *TkAGL24* flower significantly earlier than the wild type. (**D**) The transgenic plants overexpressing *TkAGL24* have significantly fewer rosette leaves compared to wild type plants. The data are presented as mean ± standard deviation (*n* = 18). ***, *p* < 0.001; ****, *p* < 0.0001 (Student’s *t* test). NC, negative control; WT, wild type. Bar = 2 cm in (**C**).

In *Arabidopsis*, MADS-box genes are a crucial class of regulatory factors that mediate floral induction and flower development, among which *AtAGL24* plays important roles in the development of floral meristems [16,18,20,21,22]. To investigate the function of *TkAGL24* on floral induction and flower development, it was overexpressed in *Arabidopsis*. A total of 13 independent T1 transgenic lines were obtained. Then, the expression levels of *TkAGL24* in the transgenic plants were verified by RT-qPCR. Three (#1, #4, and #11) out of the 13 lines, which exhibited the highest expression levels of *TkAGL24*, were selected to generate the T3 plants for further phenotypic characterization. In particular, lines #1 and #4 exhibited higher expression levels than line #11 (Figure 6A,B). The phenotypic characterization showed that the transgenic plants overexpressing *TkAGL24* flowered earlier than the wild type (WT) (Figure 6C). The further analysis of flowering time, measured by the number of rosette leaves at the bolting stage, demonstrated that the transgenic plants overexpressing *TkAGL24* had significantly fewer rosette leaves compared to the WT (Figure 6D). These results indicated that the overexpression of *TkAGL24* could promote flowering in *Arabidopsis*.

**Figure 7:**
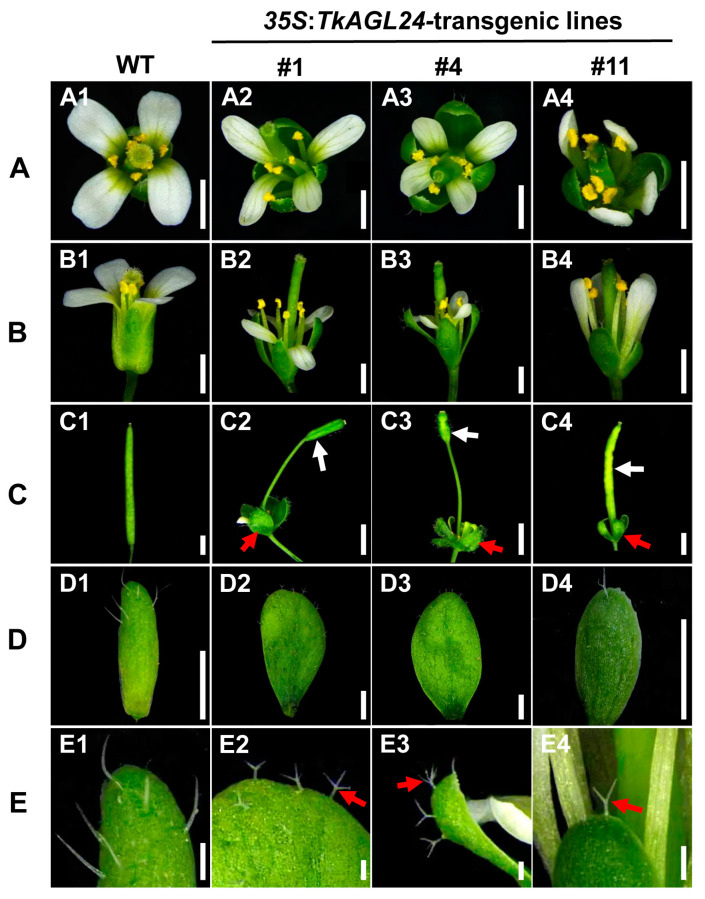
The overexpression of *TkAGL24* alters the development of floral organs in *Arabidopsis*: (**A**) The overhead-view images of the mature flowers of wild type (**A1**), transgenic lines #1 (**A2**), #4 (**A3**) and #11 (**A4**). (**B**) The side-view images of the mature flowers of wild type (**B1**), transgenic lines #1 (**B2**), #4 (**B3**) and #11 (**B4**), showing that the transgenic pistils (**B2**–**B4**) are significantly longer than the stamens, compared to those of wild type (**B1**). (**C**) The siliques in mature flowers of wild type (**C1**), transgenic lines #1 (**C2**), #4 (**C3**) and #11 (**C4**), showing the obviously defective siliques (white arrows) and leaf-like sepals (red arrows) in the transgenic lines (**C2**–**C4**). (**D**) The sepals of wild type (**D1**) and transgenic sepals (**D2**–**D4**), showing the altered shapes of the transgenic sepals (**D2**–**D4**). (**E**) The trichomes on the sepals of wild type (**E1**), transgenic lines #1 (**E2**), #4 (**E3**) and #11 (**E4**), showing the branched trichomes on the transgenic sepals (red arrows, **E2**–**E4**), compared to the unbranched trichomes on the wild type sepal (**E1**). Bars = 500 μm in (**A**,**B**), 1000 μm in (**C**), 500 μm in (**D**) and 100 μm in (**E**).

The *35S*:*TkAGL24* transgenic *Arabidopsis* plants also exhibited abnormal floral organs, in contrast to the control non-transgenic WT plants that had normal flower organs. Firstly, in the mature flowers of the transgenic plants, the pistils were higher than the petals and stamens (Figure 7A,B). This is in contrast to the WT *Arabidopsis* flowers, which had the stamens that were either longer than or equal in length to the pistils during anthesis [23,24]. Secondly, the silique development of the *TkAGL24*-overexpressing *Arabidopsis* plants was drastically affected. In particular, the *35S*:*TkAGL24* transgenic siliques were shorter and appeared morphologically abnormal compared to those in WT plants (Figure 7C). Thirdly, the transgenic sepals were transformed into the leaf-like organs that did not fall off after anthesis. In contrast to the WT *Arabidopsis* flowers, the sepals, petals, and stamens abscised after anthesis (Figure 7C). Furthermore, the sepals of *TkAGL24*-transgenic plants had branched trichomes, whereas those of the WT plants had unbranched trichomes (Figure 7D,E). Moreover, the phenotypes of lines #1 and #4, which had higher *TkAGL24* expression, were more severe than line #11 (Figure 7A–E), indicating a correlation between the expression levels of *TkAGL24* and the abnormal phenotype in the transgenic *Arabidopsis* plants. Taken together, these results suggested that the overexpression of *TkAGL24* could affect floral development in *Arabidopsis*.

### 2.6. Overexpression of TkAGL24 Caused Aberrant Ligulate Flowers in the Transgenic TKS

**Figure 8:**
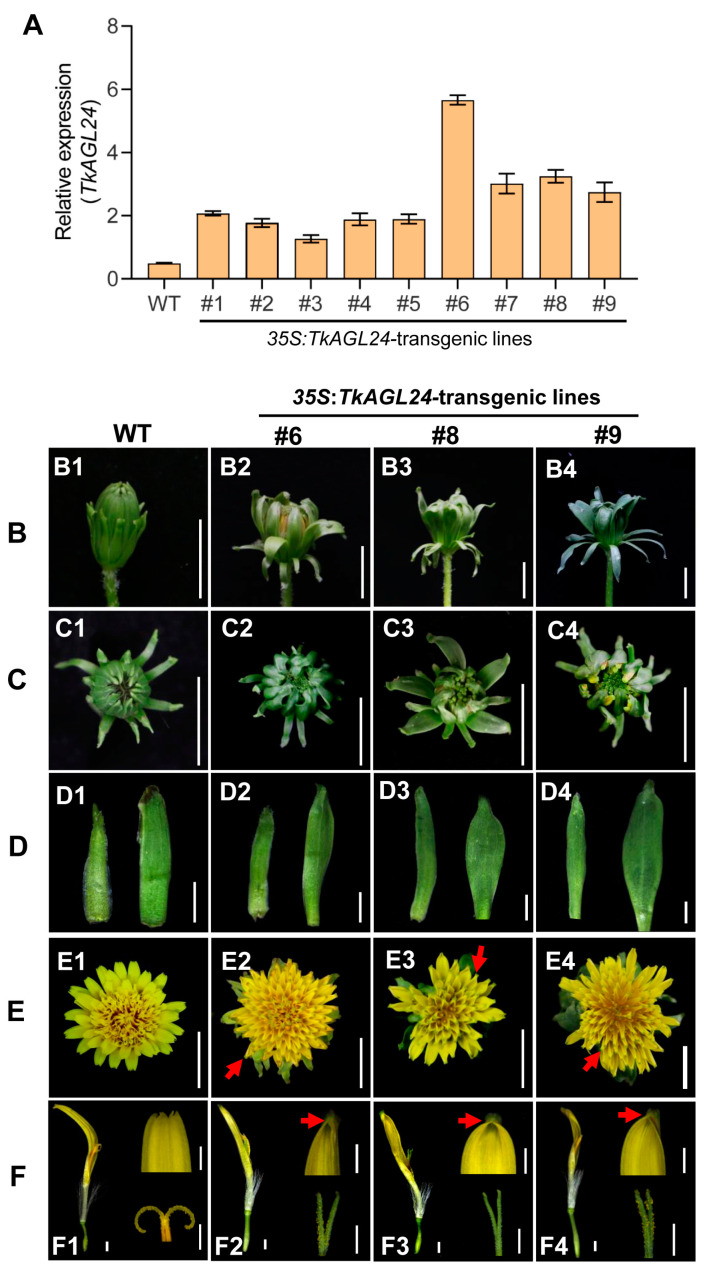
The overexpression of *TkAGL24* causes aberrant ligulate flowers in the transgenic TKS: (**A**) The relative expression of *TkAGL24* in the wild type TK20 and transgenic lines by RT-qPCR. Error bars represent the mean ± standard deviation of triplicate experiments. (**B**,**C**) The side-view images (**B**) and overhead-view images (**C**) of the unopen capitulum of TK20 (**B1**,**C1**), transgenic lines #6 (**B2**,**C2**), #8 (**B3**,**C3**), and #9 (**B4**,**C4**), showing that the transgenic involucral bracts are transformed into leaf-like structures, and the inflorescences are incompact. (**D**) The involucral bract images of TK20 (**D1**) and transgenic lines (**D2**–**D4**). The left is the outer bracts; the right is the inner bracts. (**E**) The overhead-view images of the open capitulum of TK20 (**E1**) and transgenic lines (**E2**–**E4**), showing the changed ligule morphology (red arrows). (**F**) The ligulate flower, ligule, and stigma images of TK20 (**F1**) and transgenic lines (**F2**–**F4**), showing the no-lobed ligules and green straight Y-type stigmas (**F2**–**F4**), compared to the five-lobed ligules and yellow curved Y-type stigmas in the wild type (**F1**). Bars = 1 cm in (**B**,**C**), 1 mm in (**D**), 1 cm in (**E**), and 500 μm in (**F**).

To explore the function of *TkAGL24* in TKS, the p*35S*:*TkAGL24* construct was transformed into the TK20. A total of nine independent transgenic lines were generated, all of which exhibited significantly higher expression levels of *TkAGL24* compared to the non-transgenic wild type plants (Figure 8A). Then, three transgenic lines with relatively high expression levels (#6, #8, and #9) were selected for further analysis. Due to the self-incompatibility of TKS, homozygous T1 and T2 generation plants could not be obtained. To mitigate this limitation, T0P1 (T0 generation at the first propagation) plants were generated through tissue culture to reduce chimerism and were used for further phenotypic analysis. TKS has the characteristic capitulum, which packed multiple ligulate flowers into a single head-like inflorescence and was surrounded by two layers of protective involucral bracts [25]. Compared to the wild type TK20, the *35S*:*TkAGL24* transgenic lines exhibited significant abnormalities in floral organ development. First, the involucral bracts of *TkAGL24*-overexpressing plants were transformed into leaf-like structures, with the inner bracts becoming more obvious, resulting in loosely packed inflorescences (Figure 8B–D). Second, the ligule tips of *TkAGL24*-transgenic plants failed to split into five lobes, leading to altered ligule morphology (Figure 8E,F). Third, the stigmas of *TkAGL24*-overexpressing plants became green and could not turn into the characteristic curved Y-type (Figure 8F). These results indicated that *TkAGL24* may play important roles in the development of ligulate flowers in TKS (TK20).

## 3. Discussion

This study successfully cloned the *TkAGL24* gene that encoded a MADS-box protein of 234 amino acids from TKS. TkAGL24 shared the highest homology (55.32% identity in protein sequence) with the AtAGL24 (Figure 1C), indicating that it was closely homologous to AtAGL24. Similar to the other MADS-box transcription factors, TkAGL24 had a highly conserved MADS-box domain and a moderately conserved K-box domain (Figure 3), suggesting that it is a typical MIKC-type MADS-box protein [11,26,27,28,29,30,31,32,33,34,35]. The *TkAGL24* gene exhibited a broad range of expression patterns with the highest expression levels in leaves and latex, but it was not expressed in mature flowers (Figure 4A). Taken together, our results suggest that TkAGL24 could function as a transcription factor and might play roles in growth and latex production in TKS.

The subcellular localization assay in tobacco cells using TkAGL24-eGFP showed that TkAGL24 is located in the nucleus (Figure 5A), which is consistent with its role as a transcription factor. However, the lack of transcription activation activity in the yeast system is intriguing (Figure 5B). Similar observations have been reported from the studies of other MADS-box proteins, such as AGAMOUS (AG), APETALA3 (AP3), and PISTILLATA (PI) in *Arabidopsis* [36], ZmSOC1 in *Zea mays* [37], and ZaMADS40, ZaMADS42, and ZaMADS89 in *Zanthoxylum armatum* [38], suggesting a common regulatory mechanism. These phenomena may be attributed to the diverse sequences of the C-terminal domains, which are known to act as core transcriptional activation domains in some MADS-box proteins [39,40]. In addition, TkAGL24 contains a relatively conserved motif (SDTSLKLAL) at the C-terminus (Figure 3). Given that the LXLXL motif is crucial for transcriptional repression in auxin response factors [41,42], TkAGL24 may have a transcriptional repression motif within its C-terminal domain and function by interacting with other proteins. This finding not only gives an insight into the regulatory mechanism of TkAGL24 but also provides a foundation for further exploring its interaction network in TKS.

TkAGL24 shares significant homology with AGL24-homologous proteins known to function in floral induction and flower development in other species [43,44,45]. To study the function of *TkAGL24*, we generated the p*35S*:*TkAGL24*-transgenic *Arabidopsis* plants. The overexpression of *TkAGL24* promoted flowering and caused abnormal flower organ morphology with leaf-like sepals (Figure 6 and Figure 7). Similar phenotypes have been reported for *AGL24*-homologous genes from other species when overexpressed in *Arabidopsis*. For example, the ectopic expression of *RcMADS1*, an *AGL24*-homologous gene from *Rafflesia cantleyi*, led to early flowering and leaf-like sepals and petals [46]. Similarly, overexpression of an *SVP*-like gene from perennial kiwifruit (*Actinidia* spp.) caused floral organ defects in *Arabidopsis* [47]. The molecular mechanisms underlying these phenotypes have been extensively studied in *Arabidopsis*. The AGL24 promotes flowering during the floral transition by upregulating the expression of *SOC1* and interacts with APETALA1 (AP1) to maintain floral meristem identity by directly repressing class E floral homeotic genes during the early stages of flower development [13,17]. Furthermore, our results demonstrated a potential correlation between the expression levels of *TkAGL24* and the phenotypic strength of the transgenic plants (Figure 7), consistent with the dose-dependent function of AGL24 in *Arabidopsis* [18]. These functional similarities across species highlight the conserved roles of *AGL24*-like genes in floral induction and flower development, providing a theoretical reference for elucidating the function of *TkAGL24* in TKS.

In Asteraceae plants, which exhibit the innovative capitulum structure, the functional roles of *AGL24*-homologous genes remain largely unexplored. Our study found that the overexpression of *TkAGL24* in TKS plants resulted in the transformation of involucral bracts into leaf-like structures (Figure 8B–D), a phenotype similar to that observed in p*35S*:*AGL24* transgenic *Arabidopsis* plants. This suggested a conserved role of *TkAGL24* in floral development. Interestingly, *TkAGL24*-overexpressing TKS plants also exhibited novel phenotypes in their specialized ligulate flowers, including unlobed ligules and green stigmas (Figure 8E,F), suggesting that *TkAGL24* may have species-specific complex roles in TKS, which could be different from those in other species. The finding also provided a reference for further investigation of the rarely known mechanism underlying the unique floral structures of Asteraceae plants. In addition, previous studies in rubber trees have demonstrated that HblMADS24 and HbMADS4 could regulate the expression of NR biosynthesis-related genes [48,49]. Our results showed that *TkAGL24* was expressed highly in latex (Figure 4A), implying that MADS-box genes may participate in the biosynthesis of NR in TKS.

Taken together, our results suggest that the *TkAGL24* gene, potentially a direct homolog of *AGL24* in *Arabidopsis*, plays important roles in regulating floral induction, flower development, and possibly rubber production in TKS. Although more studies of genetic mutations and biochemical functions are required for further illustrating the functions of *TkAGL24*, this study could provide a foundation for further studies on its roles in TKS and its possible application to breeding and domestication.

## 4. Materials and Methods

### 4.1. Plant Materials and Growth Conditions

The TKS germplasm line TK20 used in this study was collected from Zhaosu County in the Tekes River basin, Xinjiang. Characterized as self-incompatible, the TK20 plants were propagated by tissue culture [50]. After being grown on medium for two months, the regenerated TK20 plants were transplanted into potted soil and cultivated in growth rooms for 2–3 months under the following conditions: 24 °C, 60% relative humidity, a light 16 h/dark 8 h cycle, and 60–80 μmol/m^2^s^−1^ light intensity [50]. Then, the plants were vernalized in specialized chambers (6 °C/10 °C, 60% relative humidity, and a light 16 h/dark 8 h cycle) for four weeks. Next, they were transferred to the conventional growth rooms for further growth. The *Arabidopsis* plants used in this study were the Col-0 ecotype and grown as described previously [51]. The tobacco (*Nicotiana benthamiana*) plants were grown under the same conditions as the TKS plants.

### 4.2. RNA Extraction and Preparation of cDNA Pools

The samples from the different tissues were collected from the plants at the blooming stages, frozen in liquid nitrogen immediately, and stored at −80 °C for further use. Total RNAs were extracted from the different plant tissues using the RNAprep Pure Plant Plus Kit (TIANGEN, Beijing, China). The extraction process was carried out in strict accordance with the supplier’s instructions. Once extracted, the total RNAs were stored at −80 °C. The HiScript III 1st Strand cDNA Synthesis Kit (+gDNA wiper) (Vazyme, Nanjing, China) was used to synthesize the cDNA pools from the total RNAs as per the supplier’s guidelines. The resulting cDNA pools were then stored at −80 °C for experimental use.

### 4.3. Identification and Cloning of the TkAGL24 Gene

To identify the gene that was highly homologous to the *Arabidopsis AGAMOUS-LIKE 24 (AtAGL24*, *AT4G24540*) gene, the amino acid sequence of the AtAGL24 protein was used to perform a BLASTP search against the TK20 genome annotation database (unpublished data). The search results showed that a protein encoded by *LG06.1290* had the highest similarity to the AtAGL24 protein. Thus, we renamed *LG06.1290* as *TKS AGL24* (*TkAGL24*). Then, a pair of primers (LG06.1290-F and LG06.1290-R) was designed based on the CDS of the *TkAGL24* gene (Table S1) and used for PCR-based cloning of *TkAGL24*. The amplification of the CDS fragment was performed using the PrimeSTAR^®^ GXL DNA Polymerase (Takara, Beijing, China) following the instructions provided by the supplier. The resulting *TkAGL24* CDS fragments were purified, cloned into a T-vector, and verified by sequencing.

### 4.4. Phylogenetic Analysis of TkAGL24

To further investigate the phylogenetic relationship of the *TkAGL24* gene with the homologous genes from other plant species, the amino acid sequence of TkAGL24 was used as a query sequence to perform an NCBI BLASTP search (www.ncbi.nlm.nih.gov, accessed on 20 September 2024). Then, the amino acid sequences of the proteins from different species, which showed the highest similarities to TkAGL24, were downloaded and used to construct the phylogenetic tree using the MEGA 11 software [52] under the neighbor-joining (NJ) methods with 1000 guided replications. The accession numbers of the proteins employed in this analysis are presented in Table S2. The protein sequences of TkAGL24, TkSVP1, and TkSVP2 are listed in Table S3.

### 4.5. Expression Profile Analyses of TkAGL24

The expression patterns of the *TkAGL24* gene in the different tissues were analyzed using real-time quantitative PCR (RT-qPCR). Gene-specific primers TkAGL24-qF and TkAGL24-qR (listed in Table S1) were used. The cDNA templates from roots, peduncles, leaves, flowers, and latex samples at the full-bloom stage of TK20 were prepared as described above. The gene *Tkref* (*LG01.5808*) was used as the internal reference gene [50], and its primer sequences are also provided in Table S1. The cDNA templates were diluted 25 times. The reaction systems and amplification procedures were as described by the supplier’s instructions for the RT-qPCR kit (ChamQ Universal SYBR qPCR Master Mix, Vazyme, Nanjing, China). The reaction procedure was set as follows: 95 °C for 30 s; 40 cycles of 95 °C for 10 s, 60 °C for 30 s; and a final melt for 15 s. The relative expression levels of *TkAGL24* in different TK20 tissues were calculated by the 2^−ΔΔCt^ method.

### 4.6. Assays for Subcellular Localization of the TkAGL24 Protein

The CDS of *TkAGL24* was amplified using the primer pair TkAGL24-GFP-F and TkAGL24-GFP-R (Table S1). The amplified CDS, designed without a stop codon, was then cloned into a pCAMBIA1300-derived vector to generate the TkAGL24-enhanced green fluorescent protein (eGFP) expression cassette. Cloning was performed using the ClonExpress II One Step Cloning Kit (Vazyme, Nanjing, China) according to the manufacturer’s instructions. The resulting p*35S*:*TkAGL24*-*eGFP* construct was co-transformed with the nuclear marker construct p*35S*:*H2B-mCherry* into leaf cells of *Nicotiana benthamiana* via *Agrobacterium tumefaciens*, as described previously [53]. The p*35S*:*eGFP* construct was used as a control. After a three-day incubation period, GFP and RFP fluorescence signals were visualized using a laser-scanning confocal microscope (A1R HD25, Nikon, Shinagawa, Japan).

### 4.7. Assays for Transcription Activation Activity of TkAGL24 in the Yeast

The *TkAGL24* CDS was amplified by RT-PCR using the primer pair of pGBKT7-TkAGL24-F and pGBKT7-TkAGL24-R (Table S1). The resulting fragment was then cloned into the pGBKT7 vector to generate the construct BD-TkAGL24 (pGBKT7-TKAGL24) and transformed into *E. coli* DH5α for verification by sequencing (Nanshan Biotechnology Co., LTD., Haikou, China). Then, the BD-TkAGL24 construct, pGBKT7 (a negative control), and BD-AtMYB97 (a positive control) were transformed into the yeast strain Y2HGold, respectively. The assays were performed as described previously [54].

### 4.8. Construct of the Vector for Overexpression of TkAGL24 Gene in Arabidopsis and TKS

The CDS of the *TkAGL24* gene, including both the start and stop codons, was amplified using the primers TkAGL24-OE-F and TkAGL24-OE-R following the procedures described above. The resulting CDS fragment was then subcloned into the pCAMBIA-derived vector to generate the overexpression construct, designated as p*35S*:*TkAGL24*. The generated recombinant plasmids were further confirmed by sequencing.

### 4.9. Transformation of Arabidopsis and Characterization of the Transformants

Wild type Col-0 plants were used for transformation with the floral dip method [55]. The transformant seedlings were screened on the 1/2 MS medium supplemented with 25 mg/L of hygromycin under the condition of 22 °C and a long day cycle (16 h light/8 h dark). The selected transformants were further verified by PCR, RT-qPCR, and genetic analyses. *AtACT2* (*AT3G18780*) was used as an internal control. The phenotypic characterization of the transformant lines was performed in the T3 generation. The microscope observation was performed following the procedures described in a previous study [54].

### 4.10. Genetic Transformation of TKS and Phenotypic Analysis

The p*35S*:*TkAGL24* construct was introduced into the TK20 plants via *Agrobacterium*-mediated leaf disk transformation, following the procedure described previously [50]. A total of 258 leaf disks were used for transformation. The resulting regenerated shoots were selected for transformants on the MS medium containing 10 mg/L of hygromycin. RT-qPCR was used to confirm the selected transgenic plants. Given the self-incompatibility characteristic of TK20, the independent T0 transgenic plants were propagated to mitigate chimerism through tissue culture using the method described previously [50]. The resulting regenerated T0 plants were referred to as T0P1 (T0 generation at the first propagation) plants. Twelve T0P1 plants from three independent transgenic lines, along with wild type TK20 plants, were used for phenotypic analysis.

## 5. Conclusions

In this study, we isolated and characterized the *AGL24*-homologous gene *TkAGL24* in TKS. The *TkAGL24* gene encodes a protein consisting of 234 amino acids, which harbors the conserved MADS-box type II domain and K-box domain. *TkAGL24* was highly expressed in various tissues, with the exception of mature flowers, and its expression was not regulated by vernalization. The TkAGL24 protein was mainly localized in the nucleus and did not exhibit transcription activation activity in yeast cells. Similar to other *AGL24*-homologous genes, the overexpression of *TkAGL24* in *Arabidopsis* could promote flowering and result in aberrant flower organs. Similarly, the overexpression of *TkAGL24* in TKS affected the development of ligulate flowers. These results indicate that the *TkAGL24* gene is functionally active and could be crucial for floral induction and flower development in TKS.

## Data Availability

Data will be made available upon request.

## Supplementary Materials

The following supporting information can be downloaded at https://www.mdpi.com/article/10.3390/ijms26052271/s1.
